# Supplementary figures and images for: SARS-CoV-2 co-detection with influenza and human respiratory syncytial virus in Ethiopia: Findings from the severe acute respiratory illness (SARI) and influenza-like illness (ILI) sentinel surveillance, January 01, 2021, to June 30, 2022
Source: PLOS Glob Public Health. 2024 Apr 18;4(4):e0003093. doi: 10.1371/journal.pgph.0003093 (PMC11025837; doi:10.1371/journal.pgph.0003093)

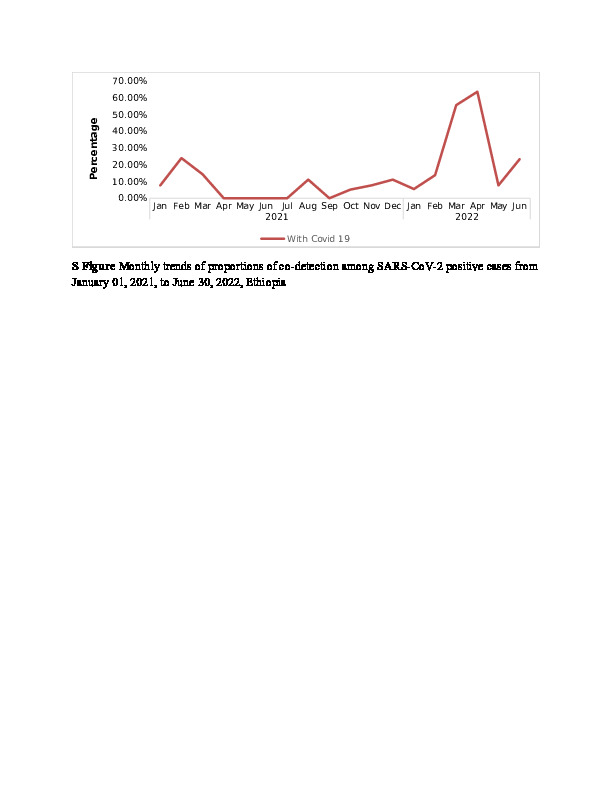

Supplement: S1 Fig — (TIF) [file pgph.0003093.s002.tif]
