# Supplementary material for: SARS-CoV-2 co-detection with influenza and human respiratory syncytial virus in Ethiopia: Findings from the severe acute respiratory illness (SARI) and influenza-like illness (ILI) sentinel surveillance, January 01, 2021, to June 30, 2022
Source: PLOS Glob Public Health. 2024 Apr 18;4(4):e0003093. doi: 10.1371/journal.pgph.0003093 (PMC11025837; doi:10.1371/journal.pgph.0003093)
Supplement: Attachment — Submitted filename: Response to Reviewers.docx [file pgph.0003093.s004.docx]

**Dear Editor:**

I appreciate the comments and suggestions of the academic editor and reviewers; their comments and suggestions were constructive and have improved our manuscript substantially. Accordingly, I have incorporated almost all of the comments and suggestions given by the academic editor and the reviewers in the manuscript. Furthermore, I have responded to each of the points raised by the reviewers as follows:

Sincerely,

Tsegaye Berkessa (Corresponding author)

**Academic Editor:**

- We noticed you have some minor occurrence of overlapping text with the following previous publication(s), which needs to be addressed:

https://www.frontiersin.org/articles/10.3389/fmed.2021.681469/full

https://bmjopen.bmj.com/content/11/11/e053768.full

https://bmcinfectdis.biomedcentral.com/articles/10.1186/s12879-020-4827-0

**Response:** Yes exactly, the overlapping text outside methodology section was edited accordingly.

- Please review your reference list to ensure that it is complete and correct. If you have cited papers that have been retracted, please include the rationale for doing so in the manuscript text, or remove these references and replace them with relevant current references. Any changes to the reference list should be mentioned in the rebuttal letter that accompanies your revised manuscript. If you need to cite a retracted article, indicate the article’s retracted status in the References list and also include a citation and full reference for the retraction notice.

**Response:** This comment has been well taken and the references were reviewed accordingly.

**Reviewer 1:**

- Ensure consistency in terminology; for instance, the manuscript refers to “SARS-COV-2” and “SARS-CoV-2”; choose one consistent term throughout.

**Response:** We appreciate your comment and corrected all the term to “SARS-CoV-2” accordingly.

- The abstract is well structured, although some refinements in language precision and consistency would enhance its clarity.

**Response:** The abstract was revised by editing inconsistent words.

- Line 100-101 there is a typographical error “real-time PCR”.

**Response:** Correction was made accordingly.

- In line 87-89, please provide specific data or references, such as the rate of co-infections.

**Response:** The comment was well accepted and replaced by specific research findings

- Consider briefly acknowledging key findings or trends from studies conducted in other parts of the world.

**Response:** The comment was accepted key findings of other studies were incorporated in the revised manuscript.

- Mention the significance of the study within the Ethiopian context. How will the findings contribute to addressing the local challenges, especially in comparison to studies conducted elsewhere?

**Response:** This comment has been well taken and revision was made accordingly.

- Consider providing a brief rationale for choosing specific variable in the regression analysis, which would enhance the understanding.

**Response:** As it was mentioned in the method section of the manuscript, we followed models building strategy steps mentioned by Hosmer and Lemeshow in applied logistic regression book. First, we used univariate analysis to identify important covariates. Secondly, all the variables with p-value < 0.25 with SARS CoV-2 co-detection in univariate analysis were fitted to the final multivariate logistic regression model.

- The shift in age categories between Table 1-2 and Table 3 raises a notable point. While Table 2 provides a detailed breakdown of age groups, the transition in Table 3 to broader categories may impact the precision of age-related findings. A transparent explanation of the rationale behind the change in categorization would strengthen the interpretation of age-related associations in the study.

**Response:** In table 1 and 2 we used detail category age following WHO classification for Influenza and all regions for the description purpose, but in binary logistic regression model (table3) less important variable categories were merged to run the regression model with the minimum rule thumb.

- In line 192, most likely this is likely a typographical error in the sentence “under two age category10”.

**Response:** The comment was accepted and corrected accordingly.

- In the multivariable analysis, the number of “yes” cases are relatively low compared to the “no” cases, raising considerations about the stability and precision of the estimated parameters. With this substantial imbalance in the number of cases between groups, there is a risk of limited statistical power, which may affect the reliability of the results and increase the potential for type II errors. The author might explore alternative methods or approaches to mitigate the impact of imbalanced groups in the analysis.

**Response:** Yes, the imbalance might affect the analysis and some age categories were modified to minimize its effect accordingly. However, most important variable categories were examined to have the lower limit of events per variable (EPV) for developing predictor model that was commonly used as a rule thumb by most researchers.

- Add a supplementary table to support the reported overall co-detection proportion (1.3%)

**Response**: The comment is accepted and supplementary table1 was added.

- In line 207, there is an extra comma 0.9%

**Response:** This definitely correct and we have modified accordingly.

- In line 208, what was the percentage for India?

**Response:** The percentage of SARS CoV-2 co-infection in the India was 0.04%. The manuscript was modified according to the comment.

- For a more comprehensive context, it would be beneficial for the authors to include the total number of inpatients and outpatients in the study. The scientific writing and English language needs editorial work.
- **Response:** In our case as it was described in the method section (case definitions) all ILI cases were outpatients and all SARI cases were inpatients. The comment was well accepted and revision of the manuscript was made.

**Reviewer #2:**

- Could you show any plots of SARI/ILI/SARS-CoV-2/influenza/RSV circulation over time?

**Response:** The comment well accepted. As the focus of this paper is mainly SARS CoV-2 co-detection we added a plot of co-detection over 18 months period in the manuscript.

- Was there a time period when the epidemics overlapped and co-infection was more common?

**Response:** We appreciate the comment and during the study period from January 01, 2021 to June 30, 2022 no epidemic were recorded but there was slight increase in number of co-detection in April month and it was the plot was added as supplementary figure 1.

- What was the seasonality of these infections over this 18-month time period?

**Response:** SARS CoV-2 co-detection plot over 18 month’s period was added in the revised manuscript.

- Did SARS-CoV-2 alter the expected seasonal timing of influenza or RSV?

**Response:** During the study period, SARS-CoV-2 didn’t alter the expected seasonal timing of influenza or RSV.

- Was there a larger flu or RSV epidemic than usual, like there was in Western countries where lockdowns meant no flu or RSV activity for much of 2020-2021?

**Response:** It was a good concern but this manuscript only focus on findings from the severe acute respiratory illness (SARI) and influenza-like illness (ILI) sentinel surveillance from January 01, 2021, to June 30, 2022. During the study period there was no larger flu or RSV epidemic than the usual trend and no lockdowns in the country.

- Did these viruses rise and fall at the same time across all 14 sites?
  **Response:** The three viral infections across 18 sites were also added as supplementary table1.
- It would be nice to have a map of the location of the study sites, shaded by population density or annotated with major cities.

**Response:** We appreciate the comment but the purpose of this manuscript is to highlight the magnitude of SARS CoV-2 co-detection with other respiratory viruses in Ethiopia from limited national SARI and ILI sentinel surveillance data. Considering, its relevance to the main objective of this manuscript and additional cost it incur, we are unable to produce scientifically sound map.

- The methods say that the assay distinguishes influenza A and influenza B. Was there enough power

**Response:** In this study, we only used influenza A/B because our focus whether SARS CoV-2 infected patient was co-infected with influenza. In addition, only limited samples were further subtyped and it lacks power for further analysis.
